# Supplementary material for: Evaluation of a Zika virus-like particle-based vaccine in a highly penetrant non-human primate model of congenital infection
Source: PLoS Negl Trop Dis. 2026 Sep 8;20(9):e0014692. doi: 10.1371/journal.pntd.0014692 (PMC13568504; doi:10.1371/journal.pntd.0014692)
Supplement: Supplementary file 1 [file pntd.0014692.s002.pdf]

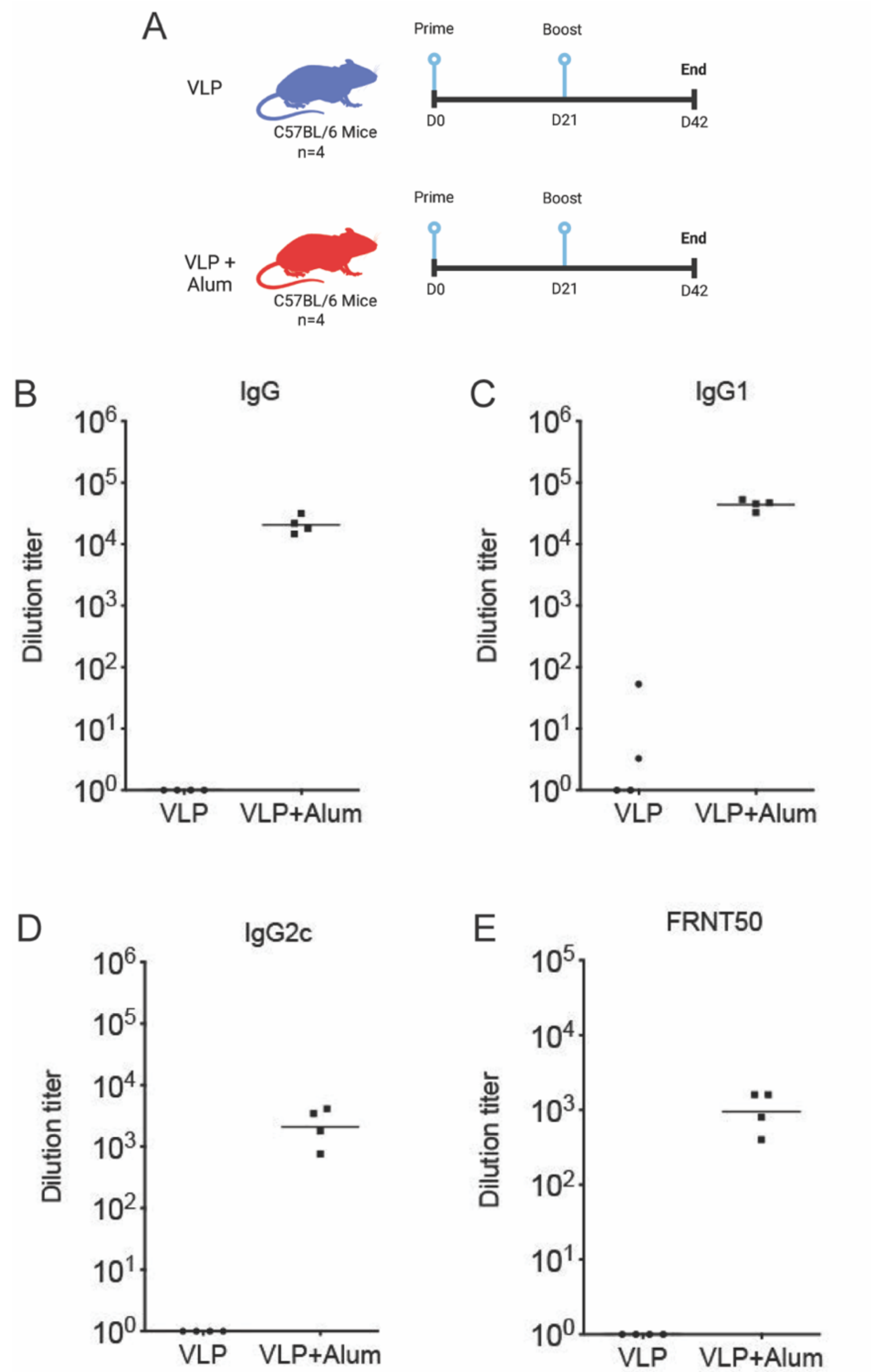

S2 Fig. ZIKV VLPs induce binding and neutralizing antibodies in C57BL/6 mouse model. ***A*,** Experimental design of C57BL/6 mouse VLP-challenge study. Mice were given two doses of 10^5 focus-forming unit equivalents determined via western blot analysis. ***B-D*,** IgG, IgG1, and IgG2c levels were determined by enzyme-linked immunosorbent assay coated with ZIKV (PRVABC59) and normalized to control serum at 42 days post-inoculation. ***E*,** The development of ZIKV-neutralizing antibodies at 42 dpi was quantified in ZIKV neutralization assays using the ZIKV-PR strain as the antigen and heat-inactivated mouse plasma at indicated time points post-inoculation. The 50% focus reduction neutralization titers ($FRNT_{50}$) were determined using non-linear regression. The LOD was a 1:50 plasma dilution and undetectable values were graphed as 0. Created in BioRender. Jaeger, H. (2026). https://BioRender.com/rl781f9
