# Supplementary material for: Evaluation of a Zika virus-like particle-based vaccine in a highly penetrant non-human primate model of congenital infection
Source: PLoS Negl Trop Dis. 2026 Sep 8;20(9):e0014692. doi: 10.1371/journal.pntd.0014692 (PMC13568504; doi:10.1371/journal.pntd.0014692)
Supplement: Supplementary file 2 [file pntd.0014692.s003.pdf]

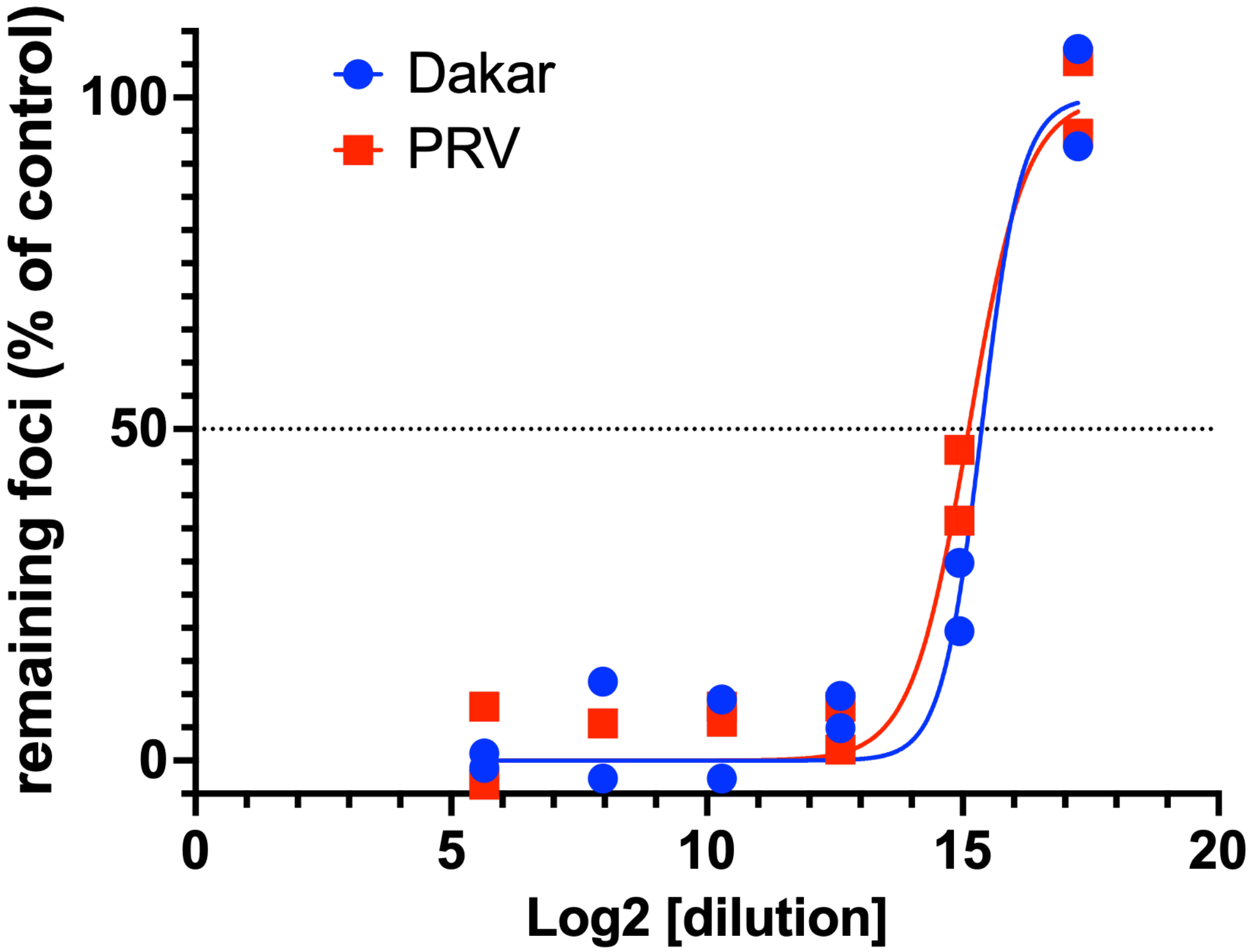

S3 Fig. Plasma from vaccinated rhesus macaque neutralizes Asian-American and African lineage ZIKV strains. ~100 ffu of an Asian- American (PRV) or African (DAK) lineage ZIKV strain were incubated with the indicated dilution of plasma collected at 78 d post-prime from VLP+alum rhesus macaque animal D5 as described in Figure 1 of the main text. The y-axis indicates the percentage of foci observed at each dilution compared to the number of foci in samples incubated with naïve plasma. The 50% focus reduction neutralization titers ($FRNT_{50}$) were determined using non-linear regression on GraphPad prism.
