# Supplementary material for: Evaluation of a Zika virus-like particle-based vaccine in a highly penetrant non-human primate model of congenital infection
Source: PLoS Negl Trop Dis. 2026 Sep 8;20(9):e0014692. doi: 10.1371/journal.pntd.0014692 (PMC13568504; doi:10.1371/journal.pntd.0014692)
Supplement: Supplementary file 3 [file pntd.0014692.s004.pdf]

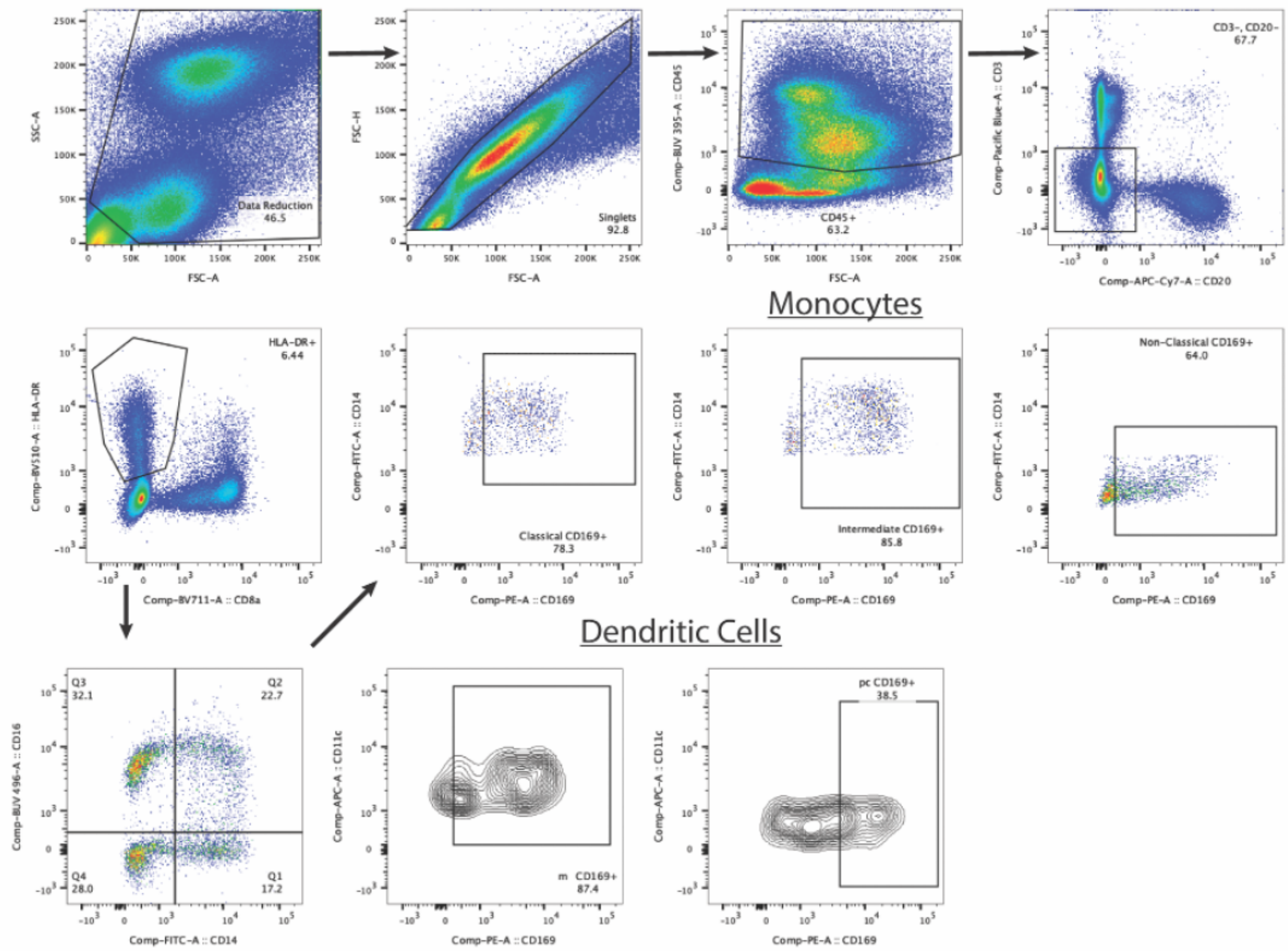

S4 Fig. Flow cytometry gating strategy for monocytes/dendritic cell panel. Monocytes and macrophages were defined as CD3-/CD20-/CD8-/HLA-DR+. Classical monocytes were further classified as CD16-/CD14+, intermediate monocytes as CD16+/CD14+, and non-classical monocytes as CD16+/CD14-. Dendritic cells were defined as CD3-/CD20-/CD8-/HLA-DR+/CD16-/CD14- with myeloid (m)DCs being CD11c+/CD123- and plasmacytoid (pc)DCs being CD11c-/CD123+. Activated cells within each subset were defined as CD169+ and set at D0 baseline for each animal. Analysis was all done on FlowJo v10.10.
