# Supplementary material for: Evaluation of a Zika virus-like particle-based vaccine in a highly penetrant non-human primate model of congenital infection
Source: PLoS Negl Trop Dis. 2026 Sep 8;20(9):e0014692. doi: 10.1371/journal.pntd.0014692 (PMC13568504; doi:10.1371/journal.pntd.0014692)
Supplement: Supplementary file 4 [file pntd.0014692.s005.pdf]

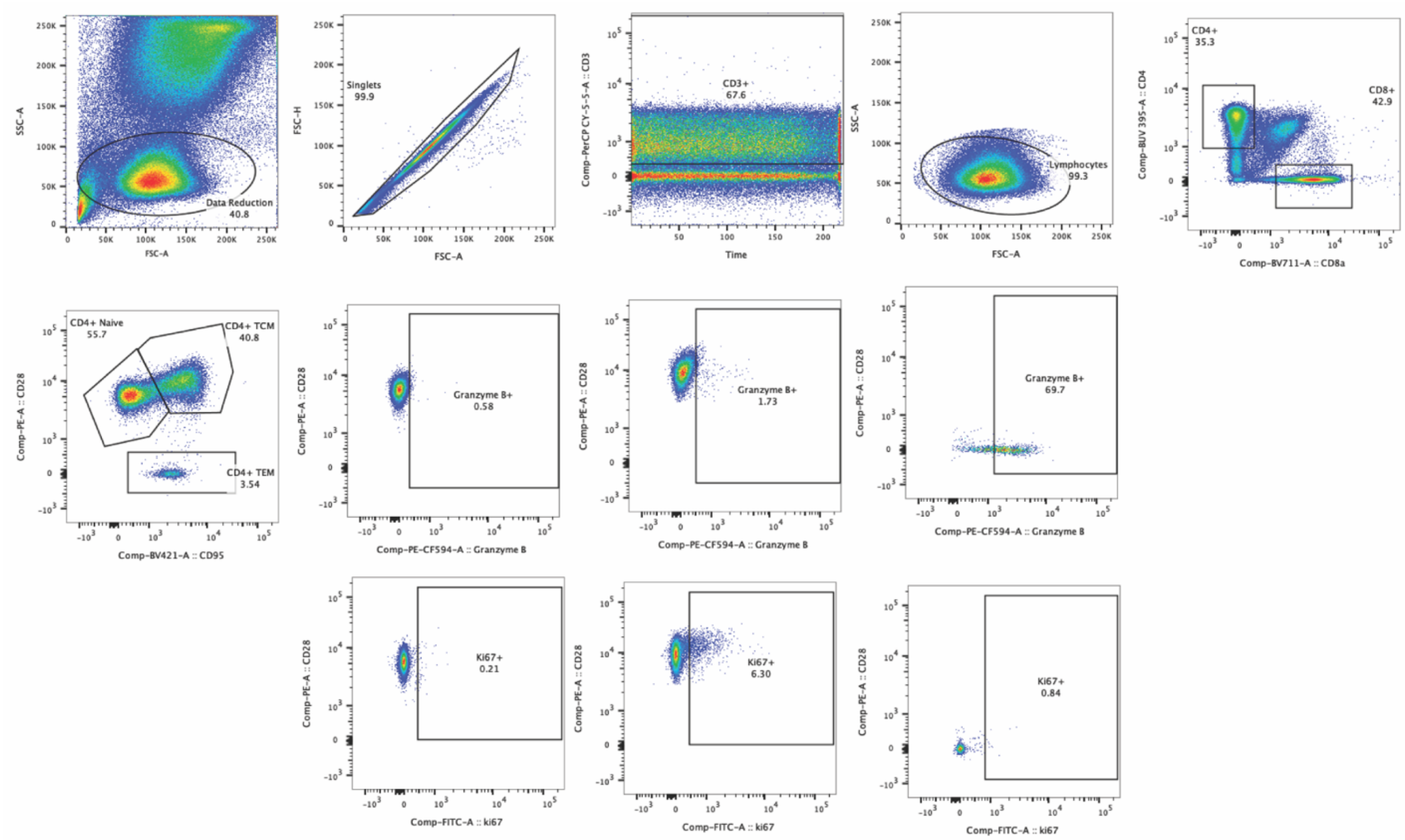

S5 Fig. Flow cytometry gating strategy for T cell panel. T cells were defined as either Naïve CD28+/CD95-, central memory were defined as CD28+/CD95+, and effector memory CD4+ or CD8+ T cells were defined as CD28-/CD95. Proliferating (Ki67+) T-cells and granzyme B expressing (granzyme B+) T-cells within each subset were quantified using these gating schemes with D0 set as baseline for each animal.
