# Supplementary material for: Evaluation of a Zika virus-like particle-based vaccine in a highly penetrant non-human primate model of congenital infection
Source: PLoS Negl Trop Dis. 2026 Sep 8;20(9):e0014692. doi: 10.1371/journal.pntd.0014692 (PMC13568504; doi:10.1371/journal.pntd.0014692)
Supplement: Supplementary file 5 [file pntd.0014692.s006.pdf]

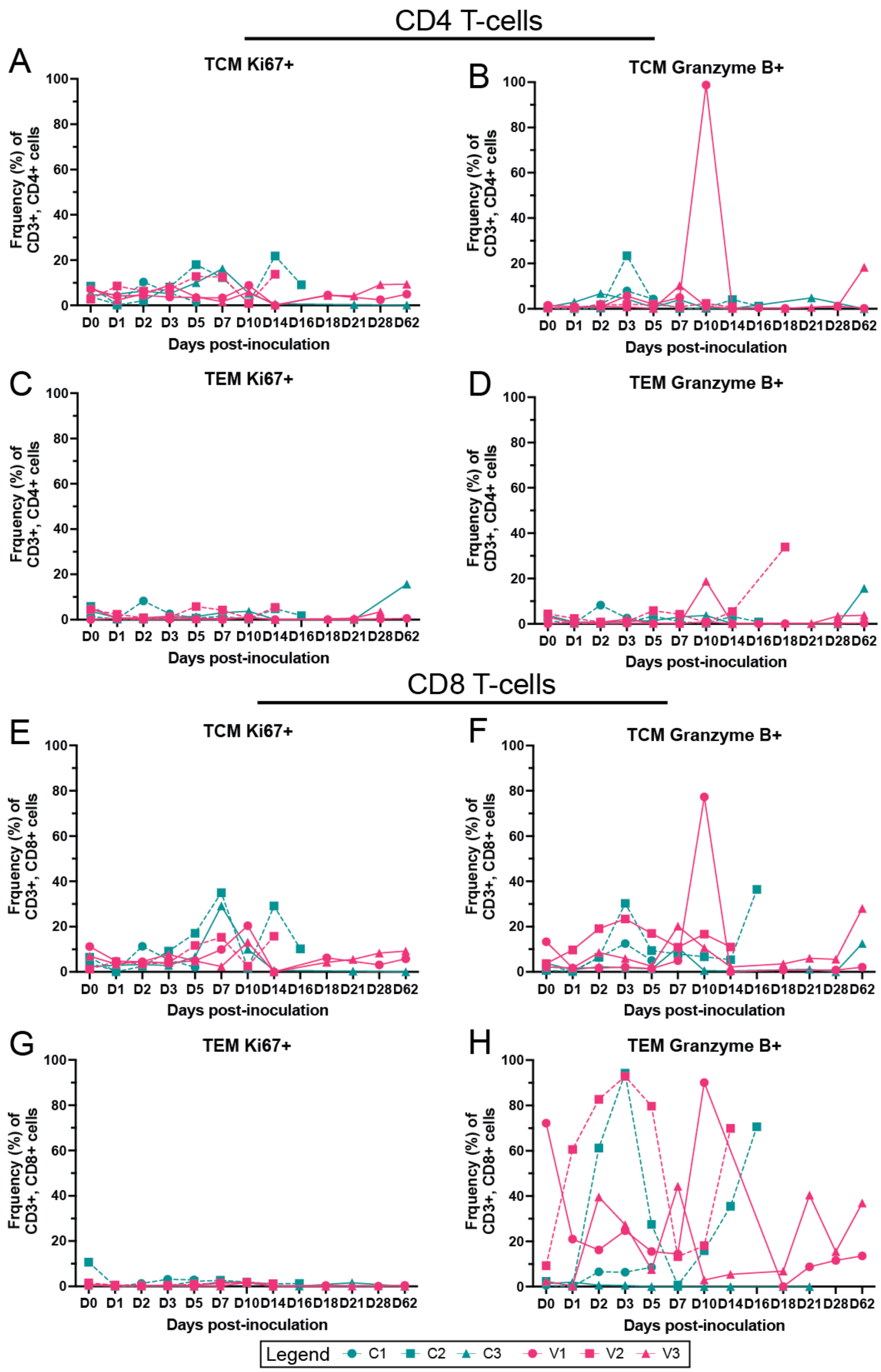

S6 Fig. Peripheral blood T cell proliferation and granzyme B expression following ZIKV challenge. Rhesus macaque PBMCs from indicated time points post-inoculation were analyzed for T cell phenotypes using flow cytometry. Proliferating central memory and effector memory CD4+ T-cell **(*A,C*)** proliferation and (***B,D***) granzyme B expression (granzyme B+) are shown for vaccinated and control cohorts. CD8+ T cells were gated similarly and are shown in ***E-H***.
