# Supplementary material for: Evaluation of a Zika virus-like particle-based vaccine in a highly penetrant non-human primate model of congenital infection
Source: PLoS Negl Trop Dis. 2026 Sep 8;20(9):e0014692. doi: 10.1371/journal.pntd.0014692 (PMC13568504; doi:10.1371/journal.pntd.0014692)
Supplement: Supplementary file 6 [file pntd.0014692.s007.pdf]

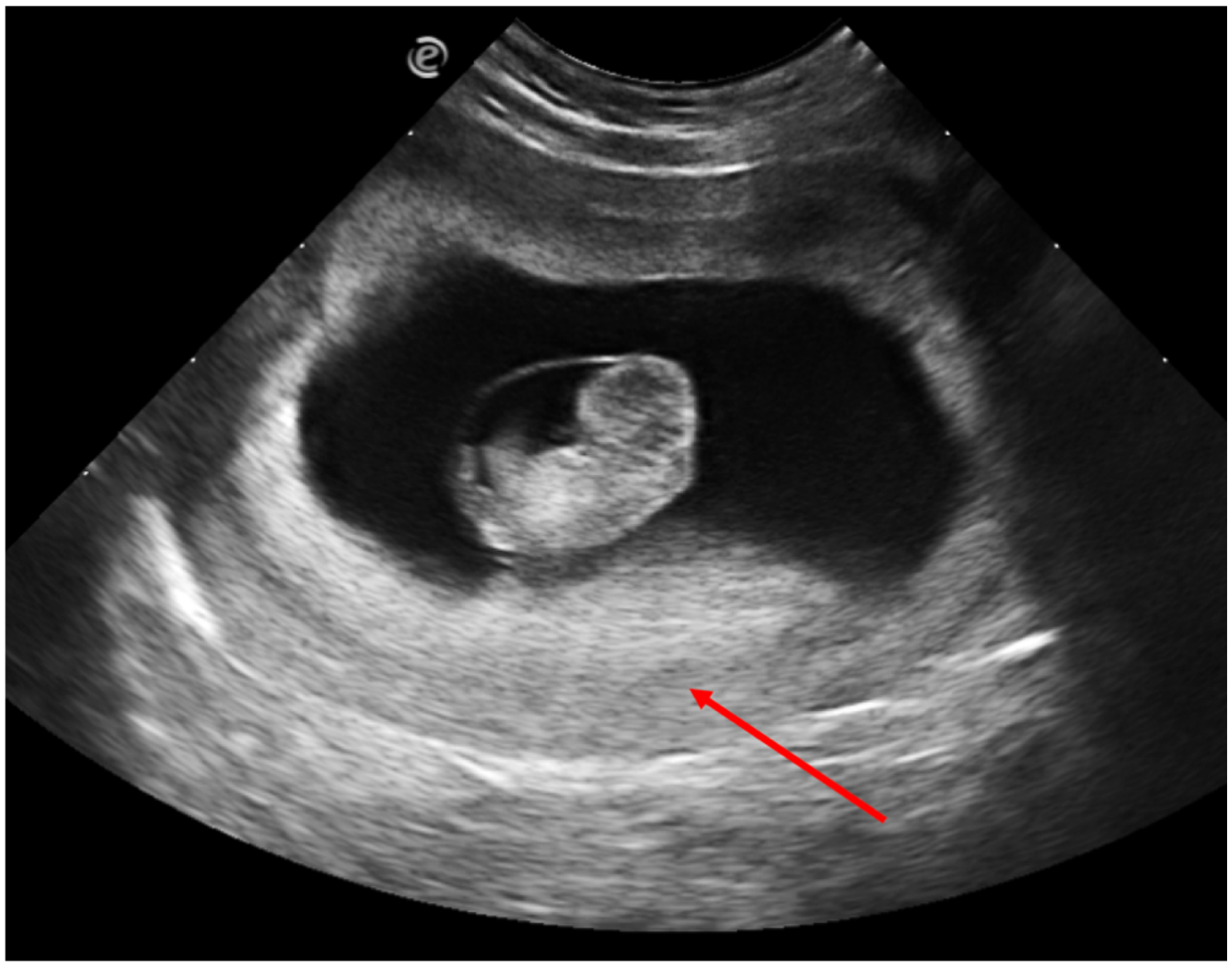

S7 Fig. Ultrasound findings in dam V3 with additional pregnancy complications. Transabdominal ultrasound imaging performed at GD35 reveals unfused amnion and chorion. Dam V3 had pregnancy-related complications prior to infection, specifically placenta previa indicated by the red arrow.
