# Supplementary material for: Evaluation of a Zika virus-like particle-based vaccine in a highly penetrant non-human primate model of congenital infection
Source: PLoS Negl Trop Dis. 2026 Sep 8;20(9):e0014692. doi: 10.1371/journal.pntd.0014692 (PMC13568504; doi:10.1371/journal.pntd.0014692)
Supplement: Supplementary file 7 [file pntd.0014692.s008.pdf]

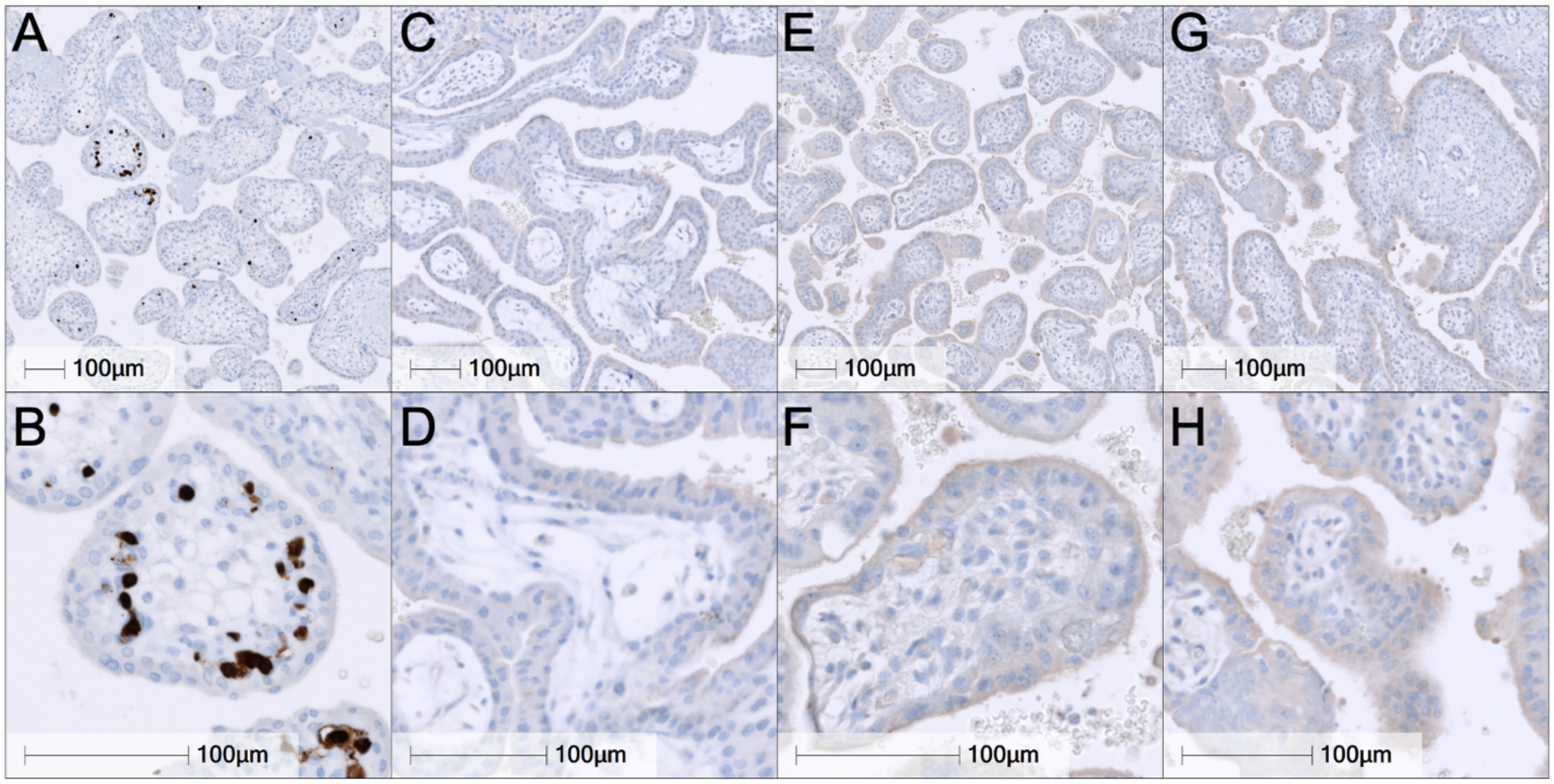

S8 Fig. Representative Immunohistochemistry of Placental Cotyledons Showing Absence of Parvovirus B19 Staining. Immunohistochemistry was performed on one cotyledon section per animal, that was 5 µm apart from the histological images scored. Sections were stained with Parvovirus-B19 specific antibody with representative images shown at low (top) and high (bottom) magnification. **A-B**, Control section from a confirmed Parvo-B19 case at low magnification **(A)** and high magnification (***B***). C-H shows negative staining for animals C1, C2, and V2 at low and high magnification for each animal. For all images, scale bars are indicated in black. Original images were taken at 20X.
