# Supplementary material for: Evaluation of a Zika virus-like particle-based vaccine in a highly penetrant non-human primate model of congenital infection
Source: PLoS Negl Trop Dis. 2026 Sep 8;20(9):e0014692. doi: 10.1371/journal.pntd.0014692 (PMC13568504; doi:10.1371/journal.pntd.0014692)
Supplement: Supplementary file 8 [file pntd.0014692.s009.pdf]

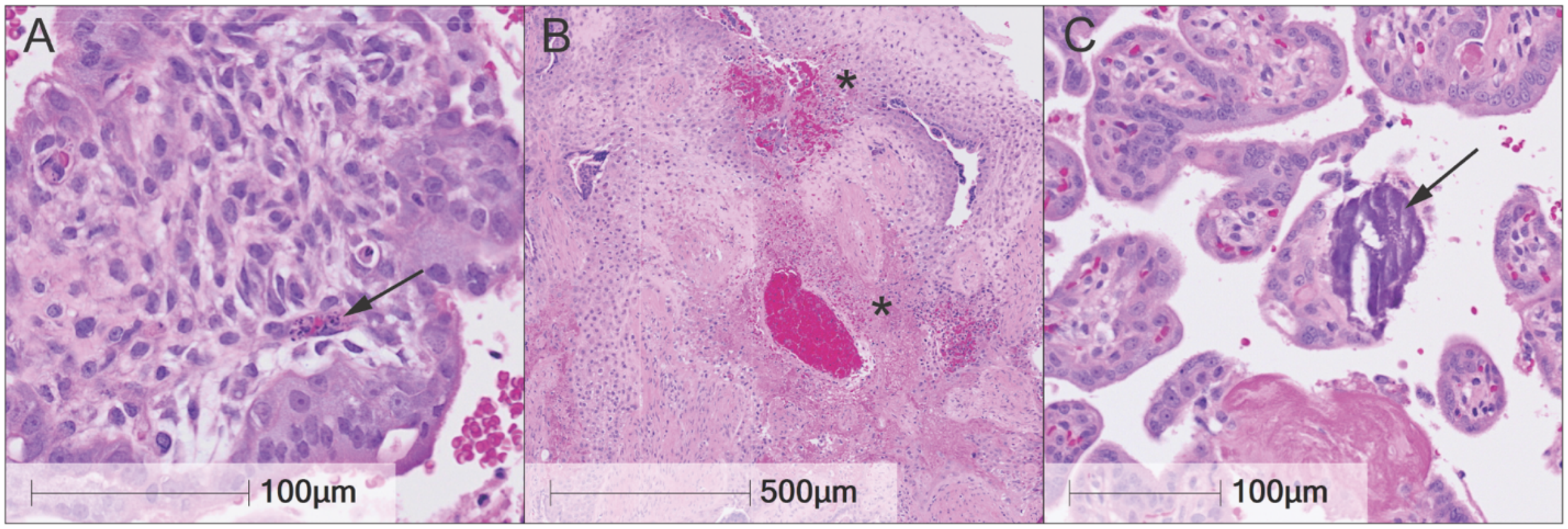

S9 Fig. Evidence of placental apoptosis, necrosis, and calcification. Representative images were scanned at 40X with Zeiss Axio scanner and the figure was created using Halo (Indica labs) with scale bars shown for each image. ***A,*** Apoptotic debris is noted by the black arrow for animal C2 which was found in several samples. ***B,*** Focal necrosis and microinfarctions are noted by the black asterisk for animal V3. ***C,*** Stromal Calcifications were seen in animal V3 and noted by the black arrow.
