# Supplementary material for: Evaluation of a Zika virus-like particle-based vaccine in a highly penetrant non-human primate model of congenital infection
Source: PLoS Negl Trop Dis. 2026 Sep 8;20(9):e0014692. doi: 10.1371/journal.pntd.0014692 (PMC13568504; doi:10.1371/journal.pntd.0014692)
Supplement: Supplementary file 9 [file pntd.0014692.s010.pdf]

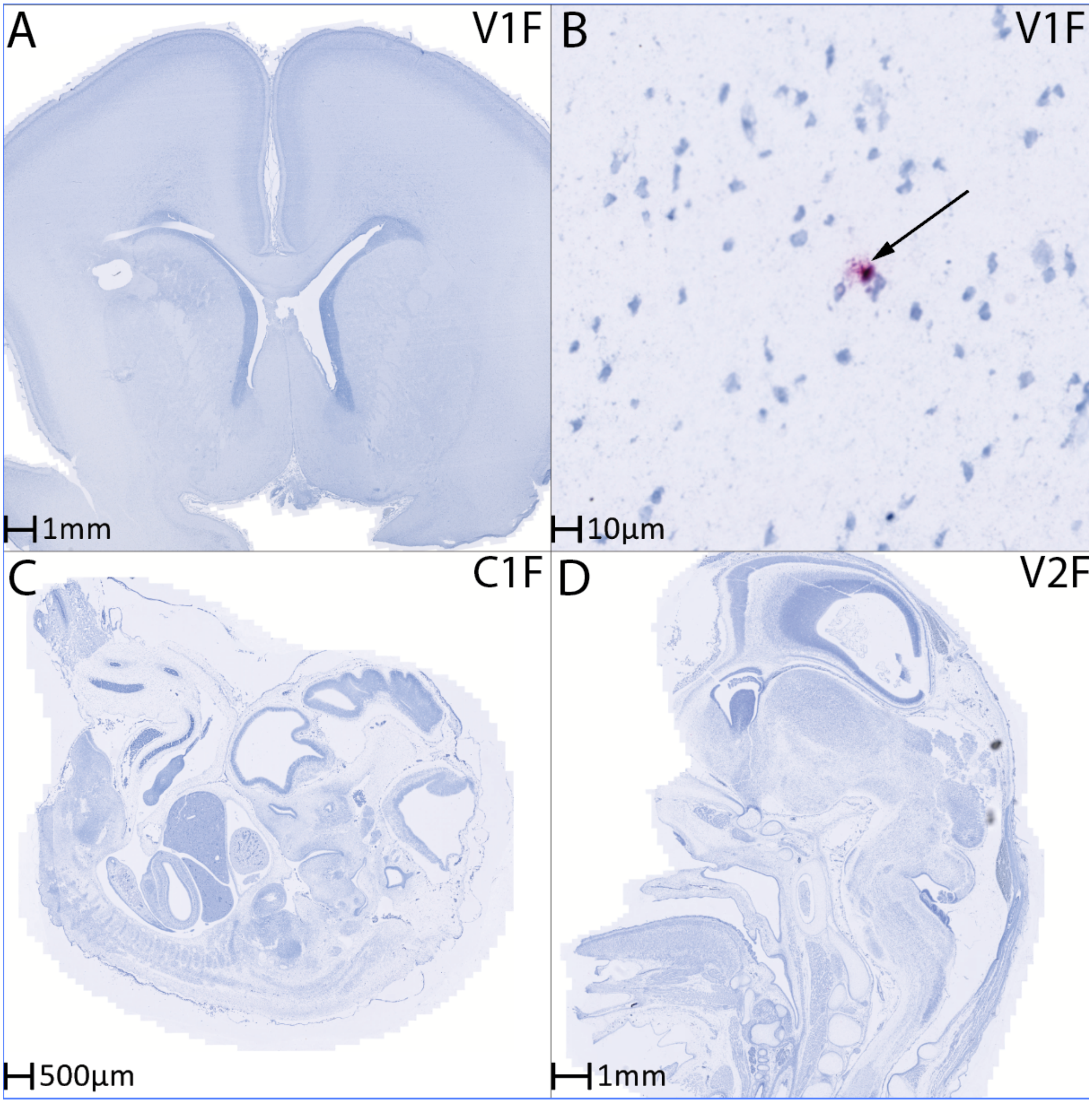

S10 Fig. Representative ISH staining demonstrating presence and absence of ZIKV RNA in brain and fetal tissue. Representative images of FFPE-blocked ***(A,B)*** V1 fetus (V1F), ***(C)*** C1 fetus (C1F), and ***(D)*** V2 fetus (V2F) stained with a ZIKV-specific ISH probe (Warp Red) and counterstained with hematoxylin and lithium bicarbonate blue. ***A, B*** Animal V1F deep brain structures (basil ganglia) at low ***(A)*** and high ***(B)*** magnification. Black arrows indicate ZIKV positivity. Representative images were scanned at 40X with a Zeiss Axio scanner and the figure was generated using Halo (Indica labs) with scale bars shown for each image.
