# Supplementary material for: Evaluation of a Zika virus-like particle-based vaccine in a highly penetrant non-human primate model of congenital infection
Source: PLoS Negl Trop Dis. 2026 Sep 8;20(9):e0014692. doi: 10.1371/journal.pntd.0014692 (PMC13568504; doi:10.1371/journal.pntd.0014692)
Supplement: Supplementary file 10 [file pntd.0014692.s011.pdf]

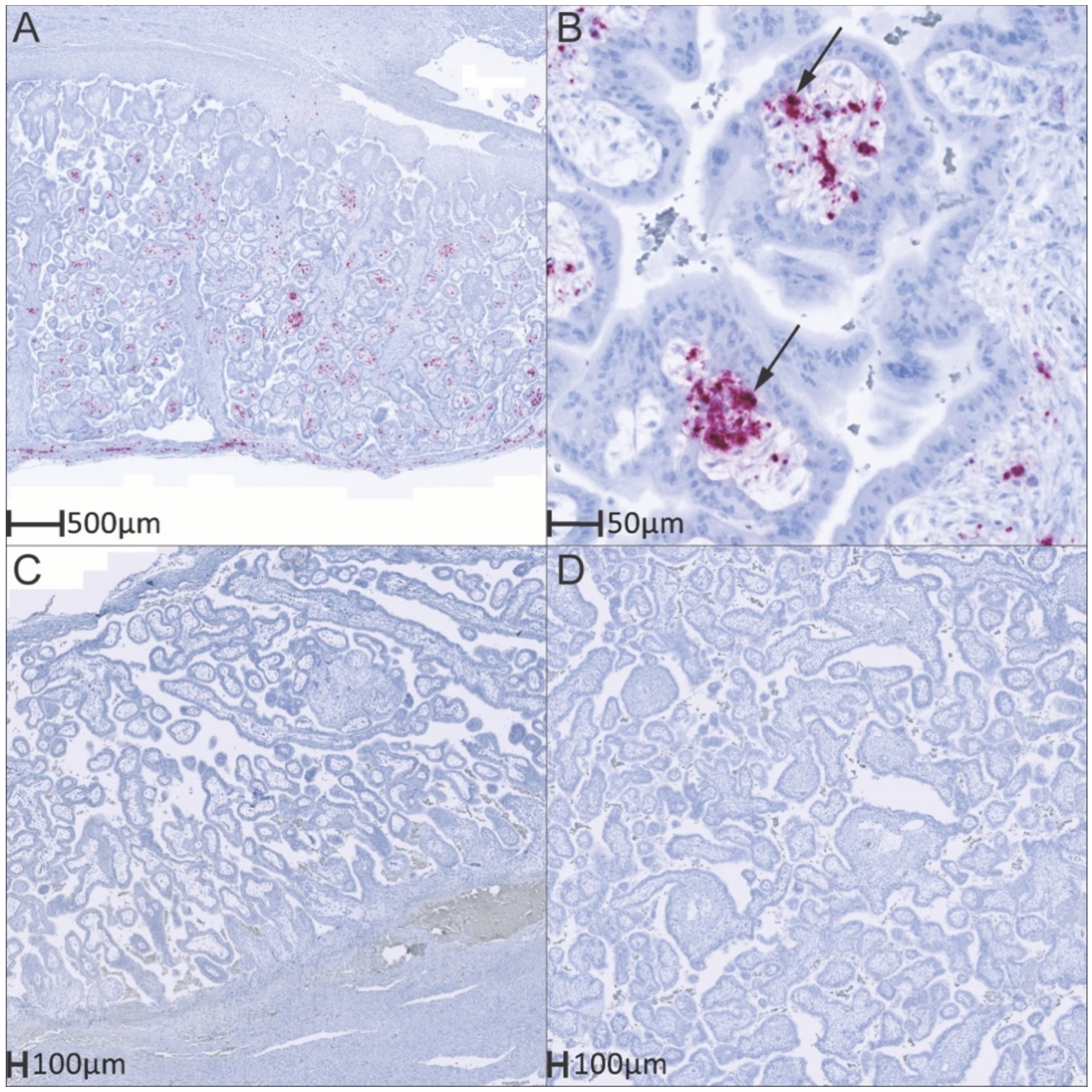

S11 Fig. Representative placental ISH staining demonstrating presence and absence of ZIKV RNA. Representative images of FFPE-blocked C2 (C2F) and V2 fetuses (V2F) stained with a ZIKV-specific ISH probe (Warp Red) and counterstained with hematoxylin and lithium blue. ***A, B*** Animal C2 full-thickness placenta at low ***(A)*** and high ***(B)*** magnification. Black arrows indicate ZIKV positivity. ***C, D*** Animal V2 full-thickness placenta at low ***(A)*** and high ***(B)*** magnification with no detected ZIKV positivity in decidua, villous or fetal membranes. Representative images were scanned at 40X with a Zeiss Axio scanner and the figure was generated using Halo (Indica labs) with scale bars shown for each image.
