# Supplementary material for: Evaluation of a Zika virus-like particle-based vaccine in a highly penetrant non-human primate model of congenital infection
Source: PLoS Negl Trop Dis. 2026 Sep 8;20(9):e0014692. doi: 10.1371/journal.pntd.0014692 (PMC13568504; doi:10.1371/journal.pntd.0014692)
Supplement: Supplementary file 11 [file pntd.0014692.s012.pdf]

Animal ID	Group	Maternal Age (years)	Mating Pairings
C1	Control	14.65	2
C2	Control	10.91	1
C3	Control	10.13	1
R1	Control	16.32	2
R2	Control	18.27	3
V1	VLP	15.23	3
V2	VLP	14.18	2
V3	VLP	11.04	1
R3	VLP	18.99	1
R4	VLP	10.27	2

S1 Table. Maternal groups and ages prior to challenge. Summary of all female Rhesus Macaques animals used in the pregnancy cohort studies and mating pairings that occurred.
