# Supplementary material for: Evaluation of a Zika virus-like particle-based vaccine in a highly penetrant non-human primate model of congenital infection
Source: PLoS Negl Trop Dis. 2026 Sep 8;20(9):e0014692. doi: 10.1371/journal.pntd.0014692 (PMC13568504; doi:10.1371/journal.pntd.0014692)
Supplement: Supplementary file 12 [file pntd.0014692.s013.pdf]

		Control (Alum)				Vaccinated (ZIKV VLP +Alum)		
	**Sample**	**C1**	**C2**	**C3**		**V1**	**V2**	**V3**
	**% vRNA+ tissues**	24/62 (38.09%)	19/63 (30.16%)	1/63 (1.59%)		2/61 (6.55%)	7/63 (11.11%)	0/59 (0%)
**Lymphoid Tissue**	Axillary LN	5.19	5.69	—		2.23	4.80	—
	Cervical LN	4.53	3.49	—			—	—
	Inguinal LN	—	3.49	—		—	2.67	—
	Mesenteric LN	6.03	5.32	—		—	4.53	—
	Popliteal LN			—				
	Retroperitoneal LN	4.72	3.04	—		—	—	—
	Sacral LN			—				
	Salivary LN	4.05	2.82	—		—	—	—
	Spleen	4.15	5.13	—		—	3.16	—
	Tracheobronchial LN					—		
	Thymus	—	—	—			—	—
	Tonsil	5.04	3.81	—		—	—	—
**Musculoskeletal**	Skin Upper Torso	4.00	—	—		—	—	—
	Triceps Brachii	2.62	—	—		—	—	—
	Biceps Brachii	1.91	—	—		—	—	—
	Brachioradialis	—	—	—		—	—	—
	Elbow	4.78	—	—		—	—	—
	Wrist	—	2.89	—		3.03	4.07	—
	Fingers	—	4.18	2.42		3.44	2.47	—
	Quadriceps Femoris	6.15	—	—		—	—	—
	Hamstring	—	—	—		—	—	—
	Knee	3.79	2.39	—		—	—	—
	Soleus	3.51	—	—		—	—	—
	Ankle	3.91	—	—		—	—	—
	Toe	—	4.35	—		—	3.83	—
**Gastrointestinal**	Parotid Gland	—	—	—		—	—	—
	Submandibular Gland	—	—	—		—	—	—
	Esophagus	2.19	—	—		—	—	—
	Stomach	4.55	4.72	—		—	—	—
	Liver	—	—	—		—	—	—
	Gall bladder	—	—	—		—	—	—
	Pancreas	—	—	—		—	—	—
	Duodenum	5.57	2.88	—		—	1.69	—
	Jejunum	4.82	—	3.50		—	—	—
	Ileum	4.80	—	—		—	—	—
	Colon	5.36	2.36	—		—	2.75	—
**Genitourinary**	Ovaries	2.93	4.24	—		—	—	—
	Fallopian Tube	—	4.06	—		—	—	—
	Uterus	2.20	3.05	—		—	—	—
	Cervix	4.15	4.78	—		—	—	—
	Vagina	2.32	4.40	—		—	—	—
	Kidney	—	—	—		—	—	—
	Urinary Bladder	—	—	—		—	—	—
	Urethra	2.24	2.19	—		—	—	—
**Cardiopulmonary System**	Heart	—	—	—		—	—	—

	Lung	—	1.96	—		—	—	—
**Endocrine System**	Adrenal Glands	4.47	—	—		—	—	—
	Mammary Gland	4.25	—	—		—	4.43	—
	Thyroid Gland	—	—	—		—	—	—
**Nervous System**								
**Central Nervous System**	Pituitary Gland	—	—	—		—	—	
	Frontal Lobe	—	1.99	—		—	—	—
	Parietal Lobe	—	—	—		—	—	—
	Temporal Lobe	—	2.09	—		—	—	—
	Occipital Lobe	—	2.57	—		—	—	—
	Basal Ganglia	4.03	2.91	—		—	—	—
	Cerebellum	—	1.85	—		—	—	—
	Brain Stem	—	—	—		—	—	—
	Cervical Spinal Cord	—	—	—		—	—	—
	Lumbar Spinal Cord	—	—	—		—	—	—
	Thoracic Spinal Cord	—	—	—		—	—	—
**Peripheral Nervous System**	Brachial Plexus	—	2.31	—		1.32	1.91	—
	Eyes*	—	1.83	—		—	—	—
	Femoral Nerve	—	—	—		—	—	—
	Dorsal Root Ganglion	3.46	—	—		—	—	—
	Sciatic Nerve	—	—	—		—	—	—
	Trigeminal Ganglion	—	—	—		—	—	—

S2 Table. ZIKV tissue distribution in dams. ZIKV RNA levels in the tissues of animals were quantified in triplicate using one-step qRT-PCR. Total RNA was generated using Trizol on precleared samples following bead beating. Approximately 70 different tissues were assessed for the presence of viral RNA in dams. Mean quantity values are presented as log10 viral copies per ml of tissue homogenate with undetectable samples indicated as **—,** and tissues not tested indicated in grey. A red intensity gradient is used to indicate vRNA levels, with dark red representing higher viral RNA quantities and lighter shades indicating lower values. Blue indicates values under the final dilution used for the standard curve, but had three copies amplified and calculated using the standard curve. calculated based on the standard curve. *Eyes are included in the peripheral nervous system since RNA is primarily extracted from the cranial nerve 2 root at the back of the retina.
