# Supplementary material for: Evaluation of a Zika virus-like particle-based vaccine in a highly penetrant non-human primate model of congenital infection
Source: PLoS Negl Trop Dis. 2026 Sep 8;20(9):e0014692. doi: 10.1371/journal.pntd.0014692 (PMC13568504; doi:10.1371/journal.pntd.0014692)
Supplement: Supplementary file 13 [file pntd.0014692.s014.pdf]

		Control (Alum)	Vaccinated (ZIKV VLP + Alum)	
**Sample**		**C3F**	**V1F**	**V3F**
Lymphoid Tissues	Axilla Region	—	—	—
	Cervical LN		—	
	Cervical region	—	—	—
	Inguinal Region	—	—	—
	Mesenteric LN	—	—	—
	Peri Pancreatic LN		—	
	Spleen	—	—	—
	Submandibular Region	2.67	—	—
	Thymus	—	—	—
	Tracheobronchial LN			—
Musculoskeletal	Ankle	—	—	—
	Upper Arm	—	—	—
	Foot	—	—	—
	Hand	—	—	—
	Lower Arm	—	—	—
	Lower Leg	—	—	—
	Upper Leg	—	—	—
	Quadriceps	—	—	—
	Soleus	—	—	—
	Thoracic Skin	—	—	—
	Toe	—	—	—
Gastrointestinal	Colon	—	—	—
	Esophagus	—	—	—
	Gall Bladder	—	—	—
	Liver	—	—	—
	Pancreas	—	—	—
	Small Intestine	—	—	—
	Stomach	—	—	—
Genitourinary	Urinary Bladder	—	—	—
	Kidney	—	—	—
	Urethra	—	—	
	Ureter		—	—
	Testes	—	—	
	Penis	—	—	
	Prostate	—	—	
	Epididymis		—	
	Male Reproductive Organ			—
Cardiopulmonary system	Aorta		—	—
	Heart	—	—	—
	Lung	—	—	—
Endocrine Systems and Exocrine Glands	Adrenal Glands	3.63	—	—
	Parotid Region	—	—	—
	Thyroid Glands	—	—	—
Central Nervous System	Cervical Spine	—	—	—
	Lumbar Spine	—	—	—
	Thoracic Spine	—	—	—
	Eyes	—	—	—

S3 Table. ZIKV tissue distribution in endpoint fetal cases. ZIKV RNA levels in the tissues of animals were quantified in triplicate using one-step qRT-PCR. Total RNA was generated using TRIzol from pre-cleared, bead-beaten tissue homogenates. Approximately 40 different tissues were assessed for the presence of viral RNA in fetuses (F). Mean quantity values are presented as log10 viral copies per µg of tissue homogenate with undetectable samples indicated as **—,** and due to the gestational age, tissues unable to be isolated for each animal are indicated in grey. A red intensity gradient is used to indicate vRNA levels, with dark red representing higher viral RNA quantities and lighter shades indicating lower values. Blue indicates values under the limit of detection calculated based on the standard curve. Fetuses C1F, C2F, V2F and brain tissues from C3F, V1F, V3F were blocked in paraffin for RNAscope analysis.
